# Tunable synthesis of SiO_2_-encapsulated zero-valent iron nanoparticles for degradation of organic dyes

**DOI:** 10.1186/1556-276X-9-501

**Published:** 2014-09-16

**Authors:** Zhou Mao, Qingzhi Wu, Min Wang, Yushi Yang, Jia Long, Xiaohui Chen

**Affiliations:** 1 State Key Laboratory of Advanced Technology for Materials Synthesis and Processing, Wuhan University of Technology, Wuhan, 430070, People's Republic of China; 2 Department of Prosthetic, School of Stomatology, Wuhan University, Wuhan, 430079, People's Republic of China

**Keywords:** Zero-valent iron nanoparticles, SiO_2_, Nanocomposites, Catalytic degradation, Organic dye

## Abstract

A series of nanocomposites consisting of zero-valent iron nanoparticles (ZVI NPs) encapsulated in SiO_2_ microspheres were successfully synthesized through a successive two-step method, i.e., the wet chemical reduction by borohydride followed by a modified Stöber method. The as-synthesized nanocomposites were characterized using X-ray diffraction, field emission scanning electron microscopy, vibrating sample magnetometer, and inductively coupled plasma-atomic emission spectrometer. The catalytic performance of SiO_2_-encapsulated ZVI nanocomposites for the degradation of organic dyes was investigated using methylene blue (MB) as the model dye in the presence of H_2_O_2_. The results showed that the degradation efficiency and apparent rate constant of the degradation reaction were significantly enhanced with increased ZVI NPs encapsulated in SiO_2_ microspheres, whereas the dosage of H_2_O_2_ remarkably promoted degradation rate without affecting degradation efficiency. The content-dependent magnetic property ensured the excellent magnetic separation of degradation products under an external magnet. This strategy for the synthesis of SiO_2_-encapsulated ZVI NPs nanocomposites was low cost and easy to scale-up for industrial production, thereby enabling promising applications in environmental remediation.

## Background

Organic dyes are used in numerous industries including textile, cosmetics, food, and pharmaceutical because of their fascinating properties, such as high wet fastness profile, brilliant shades, and relatively low cost. However, the compulsive utilization of organic dyes has caused serious environmental pollution and posed public health risks [1-3]. Most organic dyes are resistant to decompose in a natural environment and can cause serious diseases because of their transformation into genotoxic and carcinogenic species. Existing strategies focus on the removal and degradation of organic contaminants from effluents, including adsorption, coagulation, chemical oxidation, electrochemical degradation, and biological degradation, among others [4-9]. The major disadvantage of these physical methods is the dye molecules that are transferred to another phase rather than destroyed [10]. Additionally, biological degradation of organic contaminants suffers from low degradation efficiency, high cost, and rigorous degradation conditions [11]. Conversely, chemical methods display promising potential in the degradation of organic contaminants, although they require various high-performance catalysts [12-14]. The disposal of chemicals containing sludge at the end of degradation also entails complicated posttreatment processes and further use of chemicals [15]. Therefore, it is indispensable and emergent to explore the novel strategies for the degradation of organic contaminants with high efficiency and low cost.

Over the past decades, zero-valent iron (ZVI) nanoparticles (NPs) have been considered to possess promising potential in environmental remediation because of their low cost, high reactivity as a reducing agent, and ability to generating reactive oxygen species (ROS) through the Fenton reaction [16,17]. In a typical Fenton reaction, Fe^2+^/Fe^3+^ reacts with H_2_O_2_ and generates the hydroxyl radical (·OH), which is a very strong oxidant capable of decomposing various organic contaminants [18,19]. The use of ZVI powder instead of iron salts avoids the introduction of counter anions into aquatic systems. In addition, the concentration of Fe^2+^/Fe^3+^ in wastewater treated by ZVI is significantly lower than those treated with iron salts [20]. Moreover, the excess catalyst contained in the sludge could be easily recycled by magnets. However, the agglomeration of ZVI NPs is one of the most fatal shortages of this process, which results in the rapid inactivation of chemical reactivity. Therefore, modification of the surface of ZVI NPs to reduce agglomeration and prevent oxidation of ZVI NPs is necessary. Among various stabilizers, SiO_2_ coating has attracted considerable attention because of its low cost and environmental friendliness. Various strategies have been developed for the synthesis of Fe/SiO_2_ nanocomposites, including sol-gel [21-26], mechanochemical billing [27,28], spray drying [29], arc discharge [30], wet chemical route [31], and ion implantation [32]. Thus far, the low-cost and large-scale synthesis of ZVI NPs with high reactivity and mobility remains a great challenge.

In this work, a facile two-step strategy was developed to synthesize SiO_2_-encapsulated ZVI NPs. The ZVI NPs were first synthesized through wet chemical reduction with NaBH_4_ and subsequently encapsulated by SiO_2_ through the classical Stöber process. The content of ZVI NPs encapsulated in SiO_2_ was adjusted by changing the reaction parameters. The products were characterized using X-ray diffraction (XRD), field emission scanning electron microscopy (FESEM), vibrating sample magnetometer (VSM), and inductively coupled plasma-atomic emission spectrometer (ICP-AES). Moreover, the catalytic degradation of organic contaminants by SiO_2_-encapsulated ZVI NPs nanocomposites was evaluated using methylene blue (MB) as the model dye by monitoring the changes of UV-vis spectra at different time intervals at room temperature.

## Methods

### Reagents

FeCl_2_ · 4H_2_O, NaBH_4_, tetraethoxysilane (TEOS), ethanol, and H_2_O_2_ were of analytic grade (Sinopharm Chemical Reagent Co., Ltd., Shanghai, China) and used without further purification. Deionized water (16 M Ω ·cm) was obtained from a Nanopure Water Systems (Thomas Scientific, Swedesboro, NJ, USA).

### Synthesis of SiO_2_-encapsulated ZVI nanocomposites

In a typical synthesis, FeCl_2_ · 4H_2_O (0.3976 g, 2 mmol) was dissolved in 40 mL of deionized water in a 250 mL, four-necked round-bottomed flask under magnetic stirring with nitrogen atmosphere. Subsequently, NaBH_4_ (0.1139 g, 3 mmol) dissolved in another 10 mL of deionized water was added to FeCl_2_ solution under ultrasonication (FS-250 150 W, Shanghai Sonxi Corp., China). The synthesis was carried out for 30 min. The products were collected by a magnet and then washed by deionized water. The mesoporous silica coating was performed according to the modified Stöber method [33,34]. In brief, 4.5 mL of TEOS was added into 45.5 mL of ethanol, which was subsequently added into ZVI NPs with ultrasonically dispersing for 0.5 h. After that, the mixture containing ZVI NPs and TEOS was rapidly added into a mixed solution containing 9 mL of ammonium hydroxide, 16.25 mL of ethanol, and 24.75 mL of H_2_O with ultrasonically dispersing for 4 h in nitrogen atmosphere. The precipitate was collected by centrifugation (4,000 rpm, 3 min), washed alternately with deionized water and ethanol, and vacuum dried at 60°C for 4 h. In a series of syntheses, the ratio of reactants and solvents were changed. Table 1 lists the reaction parameters of the syntheses.

**Table 1:** The initial concentrations of iron salt used in the synthesis

**Number**	**Sample name**	**The initial concentration of Fe**^**2+ **^**(mmol)**
1	S-0.5	0.5
2	S-1	1
3	S-2	2
4	S-4	4
5	S-8	8

### Characterization of SiO_2_-encapsulated ZVI nanocomposites

The phase structure of the samples was characterized on a power XRD (D8 Advance, Bruker Corp., Karlsruhe, Germany) using Cu Kα radiation (*λ*) 1.5406 Å. The morphology of the samples was observed using field emission scanning electron microscope (FESEM; S4800, Hitachi Corp., Chiyoda-ku, Japan). The elemental composition was measured with an Optima 4300DV ICP-AES (Optima 4300DV, PerkinElmer Corp., Yokohama, Kanagawa, Japan). Magnetic property was measured on VSM (JDAW-2000D, Yingpu Corp., Hangzhou, China). Nitrogen (N_2_) adsorption-desorption isotherms were measured with a Micromeritics apparatus (TriStar II 3020, Micromeritics Instrument Corp., Norcross, GA, USA). UV-vis spectra were recorded on a UV-vis spectrophotometer (UV-2550 PC, Shimadzu Corp., Kyoto, Japan).

### Catalytic degradation of MB by SiO_2_-encapsulated ZVI nanocomposites

Batch experiments were carried out to evaluate the catalytic performance of SiO_2_-encapsulated ZVI NPs nanocomposites using MB as the model at room temperature. In a typical experiment, the sample (20 mg) and H_2_O_2_ (1 mL) was added into 50 mL of MB (10 mg/L) aqueous solution. The suspension was continuously stirred at room temperature under visible light irradiation. The supernatant was collected at designated time intervals for UV-vis measurement after centrifugation (10,000 rpm, 3 min). The catalytic degradation of MB was evaluated by monitoring the changes of UV-vis spectra.

## Results and discussion

### Characterization of SiO_2_-encapsulated ZVI nanocomposites

**Figure 1:**
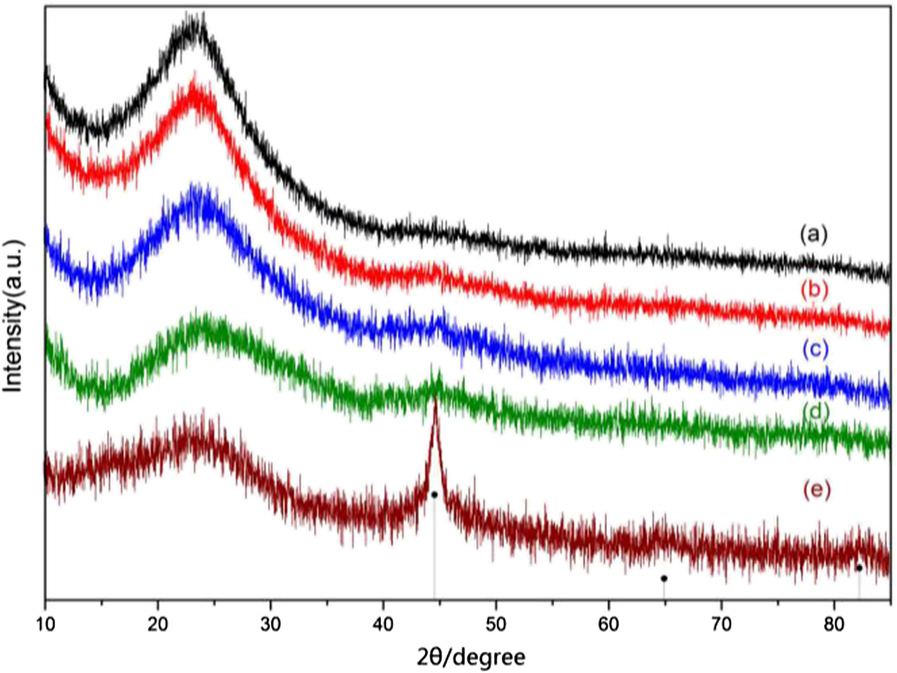
**XRD patterns of the samples synthesized with different initial concentrations of Fe**^**2+**^**. (a)** S-0.5, **(b)** S-1, **(c)** S-2, **(d)** S-4, and **(e)** S-8.

**Figure 2:**
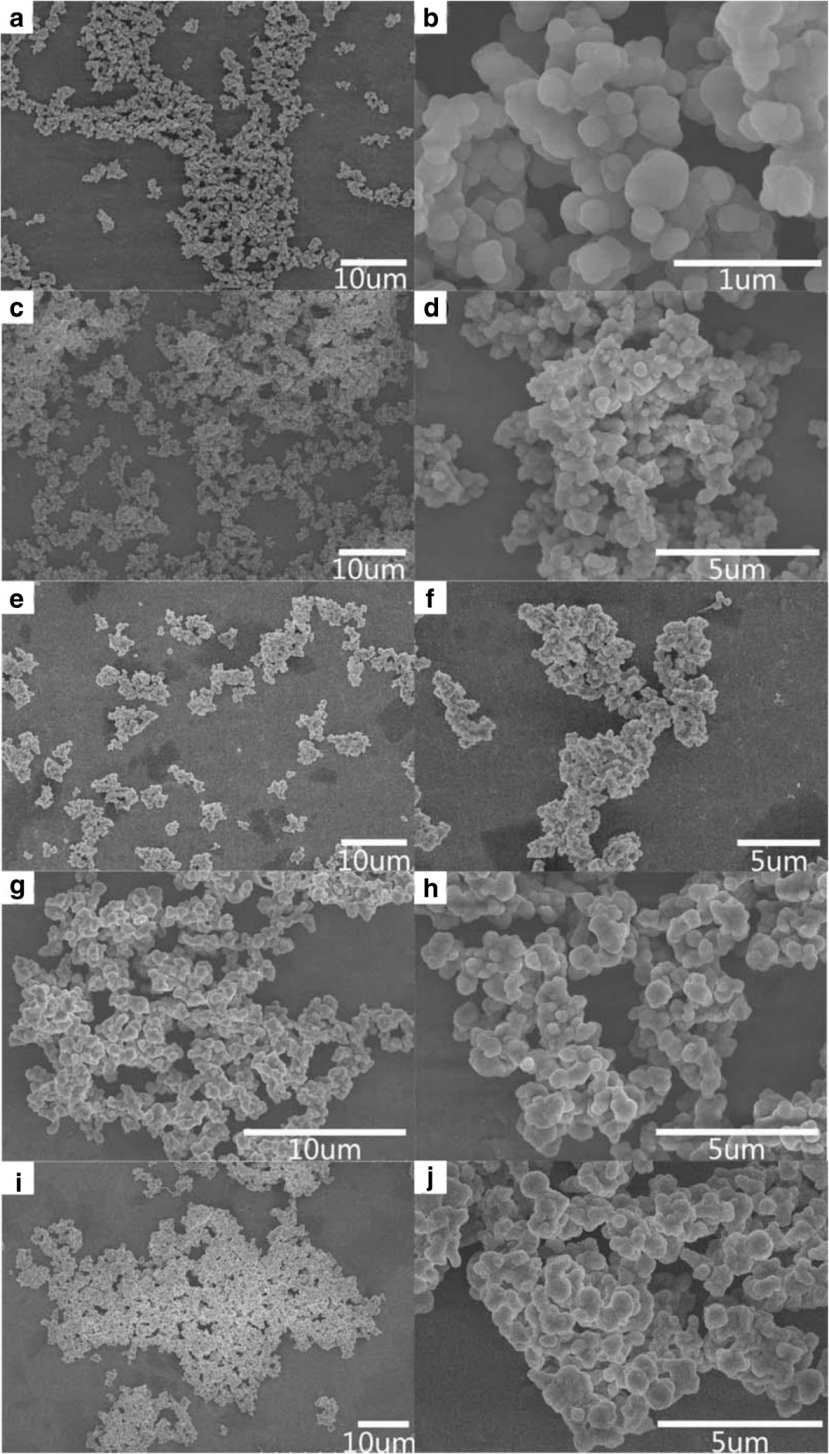
**SEM images of the samples synthesized with different initial concentrations of Fe**^**2+**^**. (a)** and **(b)** S-0.5, **(c)** and **(d)** S-1, **(e)** and **(f)** S-2, **(g)** and **(h)** S-4, and **(i)** and **(j)** S-8.

Figure 1 shows XRD patterns of the as-synthesized nanocomposites containing different contents of ZVI NPs. A broad diffraction peak at ca. 23° in the XRD patterns could be indexed to amorphous SiO_2_[35,36]. As shown in Figure 1e, the peaks at ca. 44.7°, 65.0°, and 82.3° could be assigned to Fe (JCPDS card no. 06-0696). These broad diffraction peaks indicated the small size of ZVI NPs. Notably, most of the diffraction peaks disappeared, except the peak at 44.7°, as shown in Figure 1a,b,c,d. This result was attributed to the content decrease of ZVI NPs in nanocomposites and the surface coating of SiO_2_[29]. No peak from iron oxides was observed in the XRD patterns, indicating that the ZVI NPs were well protected from oxidation because of the encapsulation by SiO_2_.Figure 2 shows the SEM images of the as-synthesized nanocomposites. Irregular spherical structures were observed, and the size of the samples significantly increased with increased ZVI NP content in nanocomposites. For example, the average size was ca. 203 ± 35 nm for the sample with the lowest ZVI NP content (S-0.5, Figure 2a,b) and ca. 725 ± 55 nm for the sample with the highest ZVI NP content (S-8, Figure 2i,j). Slight aggregations of particles were also observed with increased ZVI NPs in nanocomposites. The increase in size and the aggregation of the nanocomposites could be attributed to the increase in ZVI NP content and the magnetic interaction.

**Figure 3:**
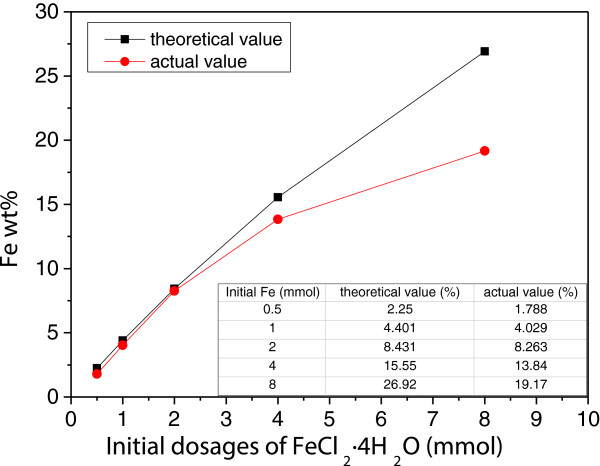
The theoretical and actual content of Fe in as-synthesized nanocomposites.

The content of Fe in the as-synthesized nanocomposites was measured by ICP analysis to identify the content of ZVI NPs in the different samples. As shown in Figure 3, the content of ZVI NPs encapsulated in SiO_2_ microsphere significantly increased with increased initial concentration of iron salts. However, the measured content of Fe in nanocomposites was consistent only with the theoretical calculation in the cases of S-0.5, S-1, and S-2. For example, in the case of S-2, the Fe content in the nanocomposite was ca. 8.3%, approximate to the theoretical content of ca. 8.4%. This result suggested that all ZVI NPs were successfully encapsulated in SiO_2_ microspheres. Meanwhile, in the cases of S-4 and S-8, the Fe content in nanocomposites was ca. 13.8% and 19.2%, relatively lower than the theoretical content of ca. 15.6% and 26.9%, respectively, suggesting that ZVI NPs were partially encapsulated in SiO_2_ microspheres. Therefore, these results demonstrated that the encapsulation ratio of ZVI NPs decreased with increased ZVI NP content in nanocomposites.

**Figure 4:**
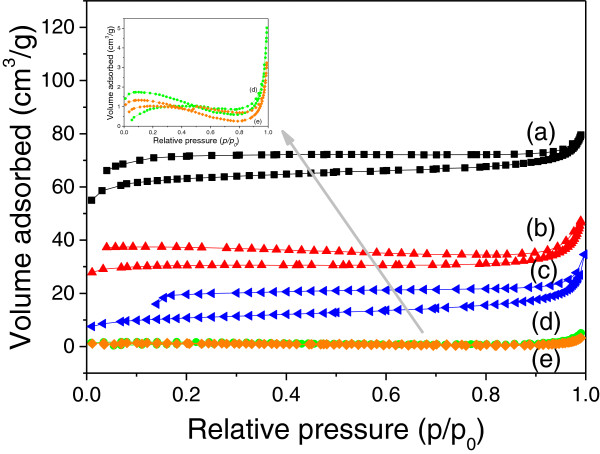
**N**_**2 **_**adsorption-desorption isotherms of as-synthesized nanocomposites. (a)** S-0.5, **(b)** S-1, **(c)** S-2, **(d)** S-4, and **(e)** S-8.

The N_2_ adsorption-desorption isotherms of the as-synthesized nanocomposites are shown in Figure 4. The isotherms of the products were indexed to type IV. It was noticeable that the isotherms of the products were not closed in the range of *P*/*P*_0_ from 0.1 to 1.0, which could be attributed to the irreversible adsorption-desorption process of N_2_ in these samples [37,38]. The specific surface areas calculated from N_2_ adsorption isotherm according to Brunauer-Emmett-Teller (BET) method were 184.3, 87.8, 33.9, 4.0, and 3.0 m^2^/g, respectively, corresponding to the nanocomposites containing different contents of ZVI NPs. The decrease of BET-specific surface areas of the nanocomposites could be attributed to the size increase of the nanocomposites, as shown in Figure 2.

**Figure 5:**
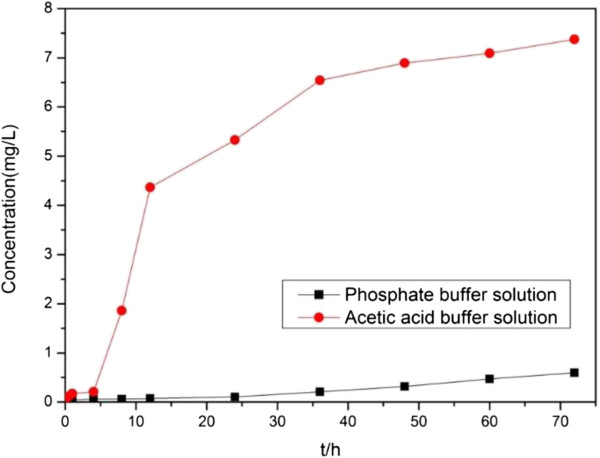
**Fe**^**2+ **^**release of S-4 in different pH environments.** Phosphate buffer solution, pH = 7.4 and acetic acid buffer solution, pH = 4.5.

The release of Fe^2+^ from the as-synthesized nanocomposites was a key factor affecting the catalytic degradation of organic contaminants. Figure 5 shows the Fe^2+^ concentration released from S-4 in different pH environments. In this test, 50 mg of S-4 was dispersed in 150 mL of buffer solution with different pH values (phosphate buffer solution (PBS), pH = 7.4; acetic acid buffer solution, pH = 4.5) and incubated at 25°C with constant shaking (130 rpm). The supernatant was collected by centrifugation (6,500 rpm, 5 min) and measured by ICP-AES. A burst release of Fe^2+^ was observed after 4 h of incubation that reached ca. 7.41 mg/mL at the end of 72 h in an acetic acid buffer solution. The released Fe^2+^ was approximately 16.0% of the content of ZVI NPs in the nanocomposite. These results indicated that the as-synthesized nanocomposites could be used as Fe^2+^ reservoir for the catalytic degradation of organic contaminants over a long period.

**Figure 6:**
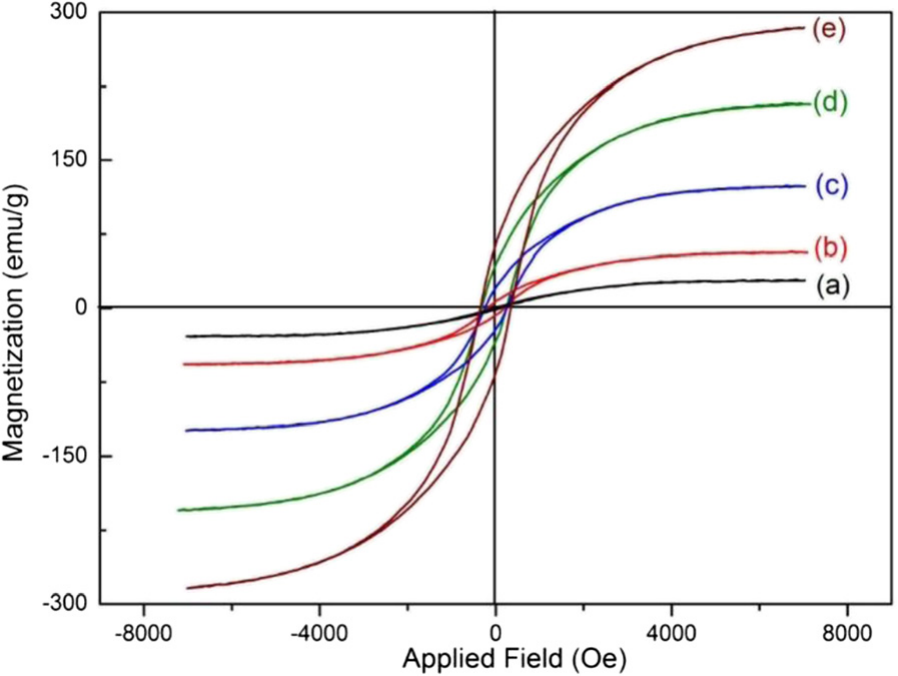
**Magnetization hysteresis loops of as-synthesized nanocomposites. (a)** S-0.5, **(b)** S-1, **(c)** S-2, **(d)** S-4, and **(e)** S-8.

The magnetic property of the as-synthesized nanocomposites was measured by VSM at room temperature. Figure 6 shows the characteristic of magnetic hysteresis loops of the as-synthesized nanocomposites, suggesting the ferromagnetic nature of the samples. As shown in Figure 6, the magnetic properties, including saturation magnetization (*M*_*s*_), coercivity, and remanence proportionally increased with increased ZVI NPs in the nanocomposites (Table 2). For example, the *M*_*s*_ values of the as-synthesized nanocomposites were ca. 28, 56, 123, 205, and 283 emu/g, corresponding to the ZVI NPs contents of ca. 1.8%, 4.0%, 8.3%, 13.8%, and 19.2%, respectively. As such, the excess catalysts could be separated and recycled by magnets.

**Table 2:** The saturation magnetization, remanence, and coercivity of as-synthesized nanocomposites

**Number**	**Sample name**	***M***_***s ***_**(emu/g)**	**Remanence (emu/g)**	**Coercivity (Oe)**
1	S-0.5	28	1.1	81.65
2	S-1	56	6.86	185.12
3	S-2	123	19.21	250.44
4	S-4	205	39.78	286.17
5	S-8	283	63.37	349.45

### Degradation assessment of MB treated with SiO_2_-encapsulated ZVI NPs

The degradation of MB was monitored by UV-vis spectrometry to evaluate the catalytic performance of the SiO_2_-encapsulated ZVI NPs nanocomposites. The changes in absorption spectra of MB solution treated with nanocomposites and H_2_O_2_ were recorded at different time intervals. The degradation efficiency (*D*) was evaluated using the following equation:

$$D\;=\;\left[\left(A_{0}-A\right)/A_{0}\right]\;\times \;100\%$$

where *A* and *A*_0_ were the absorbance at the maximum absorption peak of ca. 664 nm of MB at different time intervals and initial time, respectively.

**Figure 7:**
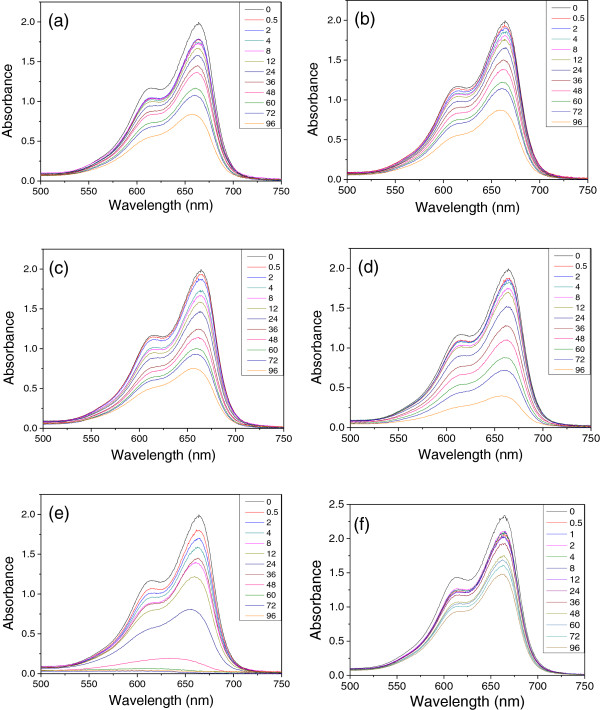
**UV-vis spectra of MB solution treated with as-synthesized nanocomposites in the presence of H**_**2**_**O**_**2**_**. (a)** S-0.5, **(b)** S-1, **(c)** S-2, **(d)** S-4, **(e)** S-8, and **(f)** pure SiO_2_ without ZVI NPs.

As shown in Figure 7, the major absorption peak of MB aqueous solution appeared at ca. 664 nm originated from MB monomer, and the shoulder peak at ca. 615 nm originated from the dimer of MB [39,40]. For comparison, MB solution was also treated with SiO_2_ microspheres synthesized under the same conditions in the absence of ZVI NPs. The significant adsorption of MB molecules by SiO_2_ microspheres was observed after 0.5 h of treatment. The adsorption efficiencies were ca. 12.7% and 36.6% after 0.5 and 96 h of treatment, respectively. SiO_2_-encapsulated ZVI NPs nanocomposites displayed significant degradation of MB in the presence of H_2_O_2_. At the end of the treatment (96 h), the degradation efficiencies of MB were ca. 59.4%, 57.1%, 64.0%, 80.9%, and 99.5%, corresponding to the as-synthesized nanocomposites with different contents of ZVI NPs. These results suggested that the content of ZVI NPs encapsulated in SiO_2_ microspheres was directly proportional to MB degradation efficiency.

**Figure 8:**
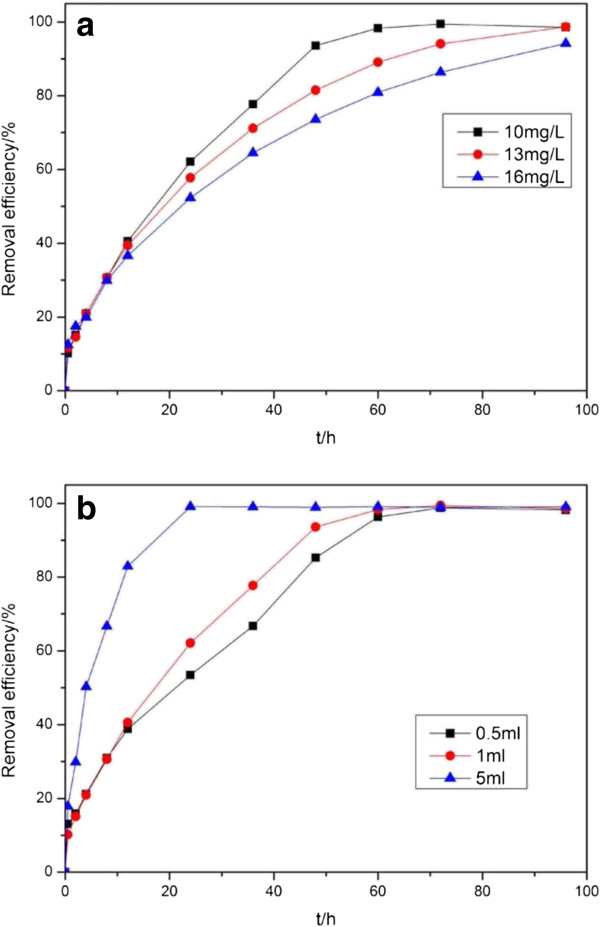
**Effects of reaction parameters on the degradation efficiency of MB by S-8. (a)** Degradation efficiency of MB with different initial concentrations and **(b)** degradation efficiency of MB in the presence of H_2_O_2_ with different dosages.

The influence of reaction parameters on the catalytic degradation of MB was further investigated. As shown in Figure 8a, MB solutions with different initial concentrations were treated with S-8 in the presence of H_2_O_2_. Results showed that, at the end of 96 h, the degradation efficiencies of MB were ca. 98.6%, 98.7%, and 94.2%, corresponding to the initial MB concentrations of 10, 13, and 16 mg/mL, respectively. Therefore, the SiO_2_ shells protected ZVI ZPs from being oxidized and prevented the adsorption of MB molecules on the surface of ZVI NPs, both of which significantly resulted in the inactivation of ZVI NP reactivity. The influence of H_2_O_2_ dosage was also studied. As shown in Figure 8b, the degradation efficiencies of the samples were ca. 53.5%, 62.1%, and 99.0% at the end of 24 h, and ca. 98.2%, 98.6%, and 99.0% at the end of 96 h, when the dosage of H_2_O_2_ was increased from 0.5 mL to 1 and 5 mL. These results suggested that increased H_2_O_2_ dosage significantly promoted degradation rate, instead of degradation efficiency.

**Figure 9:**
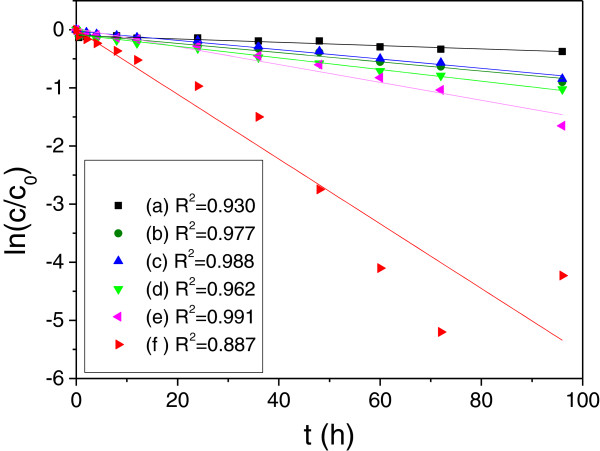
**Kinetic parameters of decoloration and adsorption performance. (a)** pure SiO_2_ without ZVI NPs, **(b)** S-0.5, **(c)** S-1, **(d)** S-2, **(e)** S-4, and **(f)** S-8.

To explore the mechanism of degradation, a pseudo-first-order kinetic model as expressed below was subsequently used to evaluate the degradation kinetic of MB by SiO_2_-encapsulated ZVI NPs [41,42]

$$\ln\;\left(c_{t}/c_{0}\right)\;=\;-k_{\mathrm{obs}}t$$

where *c*_0_ is the initial concentration (mg/L) of MB in solution, *c*_*t*_ is the concentration of MB at reaction time *t*, and *k*_obs_ is the apparent kinetic rate constant of the pseudo-first-order reaction model. Figure 9 shows the linear relationship by plotting ln (*c*/*c*_0_) against *t* (*h*). The apparent kinetic rate constant (*k*_obs_) calculated from the slope of the regression lines were ca. 0.00793 min^−1^ for S-0.5, 0.00801 min^−1^ for S-1, 0.01001 min^−1^ for S-2, 0.01537 min^−1^ for S-4, and 0.05571 min^−1^ for S-8, compared with 0.00287 min^−1^ for SiO_2_ microspheres without ZVI NPs. These results indicated that the degradation reaction was enhanced with increased ZVI NP content in nanocomposites.

In general, ZVI in the presence of H_2_O_2_ produces a Fenton-like reaction through generating numerous · OH, which can destroy various organic contaminants (such as halogenated hydrocarbons, aromatic compounds, detergents and pesticides, etc.) [16-19]. In the present study, ZVI NPs were encapsulated in SiO_2_ nanosphere, preventing from the surface oxidation of ZVI NPs. Meanwhile, ZVI NPs acted as Fe reservoir releasing iron ions (as shown in Figure 4), which catalytically broke down the H_2_O_2_ molecules into · OH. It was suggested that · OH can attack the C-S^+^ = C and C-N = C group in MB molecules, resulting in the split of the S^+^ = C and N = C double bond [43,44]. The further attack of · OH on S-Cl, C-NH_2_, and C-SO_3_H containing in various intermediates finally generated inorganic molecules or structures, such as H_2_O, CO_2_, Cl^−^, NO_3_^−^, and SO4^2−^.

**Figure 10:**
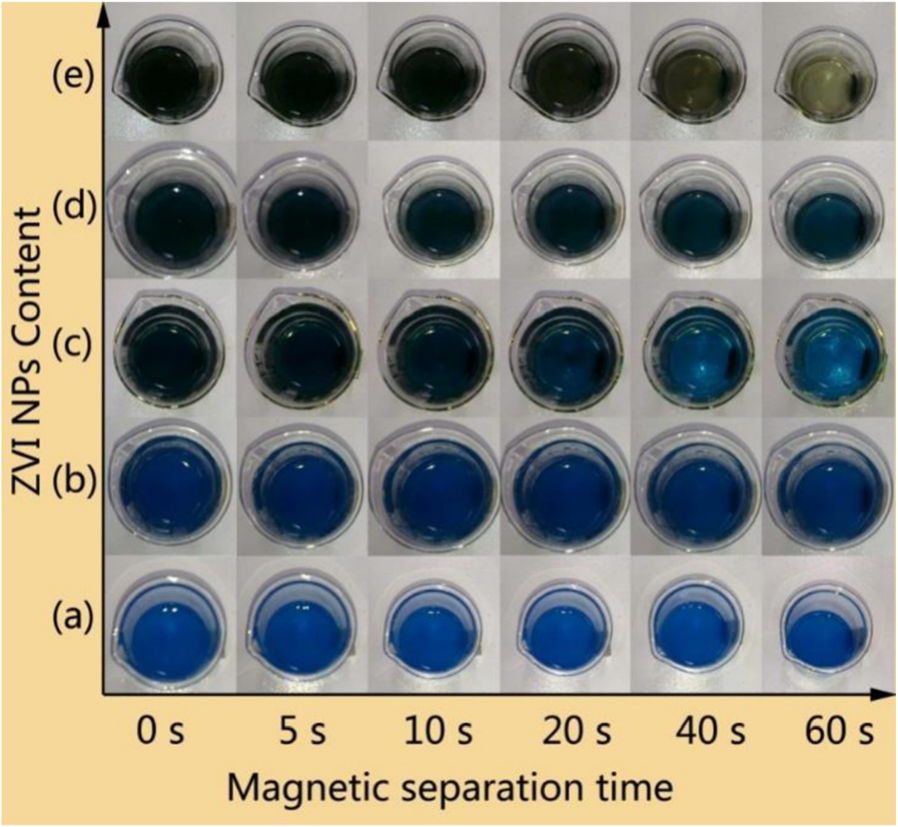
**Magnetic separation of MB solution treated with as-synthesized nanocomposites for 96 h using an external magnet. (a)** S-0.5, **(b)** S-1, **(c)** S-2, **(d)** S-4, and **(e)** S-8.

A large amount of sludge is usually produced in the coagulation phase after wastewater treatment by various catalysts, resulting in complicated posttreatment processes [45,46]. Accordingly, the magnetic separation of the as-synthesized nanocomposites in treated MB solution was used to study the recycling and separation of catalyst. Figure 10 shows the magnetic separation of the as-synthesized nanocomposites containing different contents of ZVI NPs from MB solution after 96 h of treatment. No obvious change was observed when the MB solution was treated with S-0.5 and S-1 in the presence of H_2_O_2_ at the end of 96 h. This result could be attributed to low content of ZVI NPs in the nanocomposites and low degradation of MB. However, when the dye was treated with S-2, S-4, and S-8 in the presence of H_2_O_2_, obvious decoloration and magnetic separation of sludge were observed. For example, in the case of S-8, the muddy solution became nearly clear within 1 min under exposure to an external magnet (Figure 10e). These results demonstrated the excellent performance of SiO_2_-encapsulated ZVI NPs nanocomposites in the catalytic degradation of organic dyes and magnetic separation of degradation products.

## Conclusions

In summary, SiO_2_-encapsulated ZVI NPs nanocomposites with tunable Fe content were synthesized using a two-step strategy. The results demonstrated that the catalytic degradation of MB depended on the content of ZVI NPs encapsulated in SiO_2_ microspheres. The dosage of H_2_O_2_ significantly promoted degradation rate without changing degradation efficiency. Furthermore, the content-dependent magnetic property of ZVI NPs enabled easy separation of the degradation products from the coagulated sludge using an external magnet. This strategy was facile and low cost for the industrial production of ZVI NPs for applications in environmental remediation.

## Competing interests

The authors declare that they have no competing interests.

## Authors’ contributions

ZM carried out the synthesis and characterization of SiO_2_-encapsulated ZVI nanocomposites. MW, YY, and JL participated in the measurement of degradation of MB by nanocomposites. ZM and QW analyzed the data and drafted the manuscript. XC and QW designed the whole work and revised the manuscript. All authors read and approved the final manuscript.
